# Handling of Breast Milk by Neonatal Units: Large Differences in Current Practices and Beliefs

**DOI:** 10.3389/fped.2018.00235

**Published:** 2018-09-04

**Authors:** Daniel Klotz, Stefanie Jansen, Corinna Gebauer, Hans Fuchs

**Affiliations:** ^1^Department of Neonatology, Center for Pediatrics, Medical Center - University of Freiburg, Faculty of Medicine, University of Freiburg, Freiburg, Germany; ^2^Department of Neonatology, University Children's Hospital, Leipzig, Germany

**Keywords:** bacterial contamination, cytomegalovirus, breast milk, infant, pasteurization, premature, mothers own milk

## Abstract

**Background:** Breast milk (BM) for premature infants is subjected to multiple steps of processing, storage and distribution. These steps may influence the quality and safety of BM. Guidelines concerning the use of mother's own milk are either not available or limited to specific aspects of BM handling and are based on evidence of variable strength. This may result in diverse BM handling routines by health care professionals.

**Objective:** We surveyed neonatal units to increase the knowledge about the current practice of BM handling routines of mother's own milk and to identify controversial aspects that could give directions for future research.

**Methods:** An online-based questionnaire was sent to 307 different neonatal departments providing level III to level I neonatal care within Germany, Austria and Switzerland. Practices concerning screening for cytomegalovirus and BM bacteria, pasteurization, fortification, storage, workforce and the incidence of BM administration errors were surveyed.

**Results:** A total of 152 units, 56% of contacted level III units and 51% of level II units, participated in the survey (Germany 53%, Switzerland 71%, and Austria 56%). We found differences concerning indication and method of CMV inactivation (performed by 58%), bacterial count screening (48%) and bacterial count reduction (17%) within participating units. Thirty different thresholds for bacterial BM counts were reported by 65 units, resulting in pasteurization or discarding of BM. The use of nutrient analysis (12%) and fortification regimens in addition to standard multicomponent fortifiers (58%) using either individual (93%), targeted (3%), or adjusted (4%) fortification protocols varied profoundly. There is a high variability in staff and available facilities for BM handling. 73% of units report about BM administration errors.

**Conclusion:** There is a wide variability in most aspects of BM handling in the participating units. Despite limited evidence labor and cost intensive procedures are applied which may have an impact on BM quality.

## Introduction

Mothers own breast milk (BM) is the preferred source of nutrition for the term and preterm infant (1). However, certain aspects must be considered when feeding BM to premature infants: Viruses, such as cytomegalovirus (CMV), and bacteria are transmitted via BM and may prompt BM treatment (2, 3). BM for the preterm infants needs to be expressed, collected and, depending on the individual organizational structures of the neonatal unit, transported to a designated site for further handling or storage. Upon distribution to the neonatal ward the milk needs to be (re)labeled, fortified to meet the nutritional demand of the preterm infant and reheated before it can finally be fed to premature infant (4).

These BM handling routines may be hazardous to its quality and safety (5). Hence, departmental organizational structures and operational procedures that ensure optimal BM handling and treatment need to be in place (6). However, there is a paucity of evidence-based data concerning optimal BM handling (5). Consequently, existing recommendations are based on evidence of very variable strength and this may result in diverse BM handling practices by health care professionals (7). Few data are available about the current approaches of neonatal departments for handling of mothers own milk (8–10).

The aim of this cross-sectional survey was to describe current practices of BM handling routines of mother's own milk within neonatal units and to identify controversial aspects of BM treatment that may merit further research for guiding daily clinical practice on the neonatal ward.

## Materials and methods

A structured and stratified online-based questionnaire was sent to 307 different neonatal units within Germany (*n* = 259), Austria (*n* = 34) and German speaking Switzerland (*n* = 14) between June 8th 2016 and March 1th 2017 using an online survey tool (SurveyMonkey, Portland, OR). We aimed to include all neonatal units within the participating countries, identified via the respective national neonatal and/or pediatric society or internet research. We assessed the level of neonatal care and the number of very low birth weight infants per unit per year. The screening rate for maternal CMV serostatus, the unit specific indications, methods and threshold levels for CMV inactivation and/or bacterial count reduction were surveyed. The feeding regimen for preterm infants according to the maternal CMV serostatus, bacterial BM count and postmenstrual age were inquired. Furthermore, we asked to detail the strategies for BM fortification, the prevalence and applied techniques for BM nutrient analysis as well as the condition of BM storage, departmental organizational structures and allocated staff for BM handling. The questionnaire is available as Supplementary Material. Statistical analysis was performed using GraphPad Prism (V5.02, GraphPad, San Diego, CA). Categorical variables are presented in absolute numbers and percentages. Percentages apply to the number of answers for any given question. We reported quantitative data as mean and standard deviation or median and interquartile range (or range) where applicable.

## Results

We received a total of 152 replies. Fifty-six percent of the 189 contacted units that provided level III and 51% of the 75 units that provided level II of neonatal care (definition according to the American Academy of Pediatrics) participated in the survey. Response rate per country was 53% for Germany, 71% for Switzerland and 56% for Austria. Of the 43 contacted well baby units (level I) only eight returned the questionnaire. The median number of very low birth weight infants for level III units was 54 (IQR 36-79).

### Cytomegalovirus screening and inactivation

**Figure 1:**
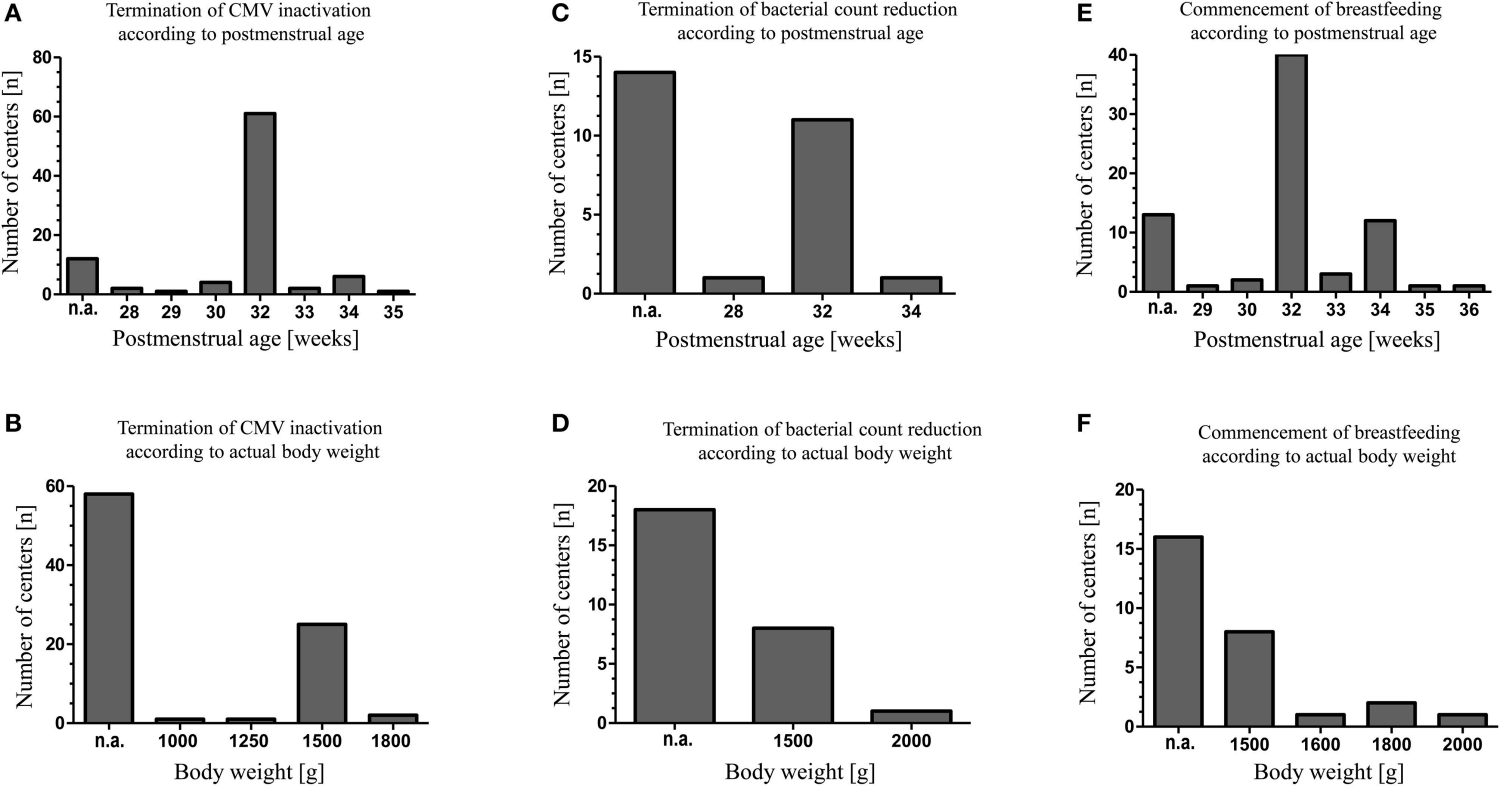
Indications of individual neonatal units for CMV inactivation, reduction of bacterial breast milk count and initiation of breastfeeding in case of maternal CMV seropositivity. The decisions to commence breastfeeding, to terminate CMV inactivation or bacterial count reduction were made either depending on the infants postmenstrual age **(A,C,E)** or depending on the infants' actual body weight **(B,D,F)**. N.a., respective criteria were not applied.

Maternal CMV screening was performed by 87 (85%) of level III units and by 25 (63%) of level II units. Untreated raw colostrum of CMV seropositive mothers was fed by 57 units (66%) for a median of 4 days (range 2–10). Thereafter, CMV inactivation using Holder-Pasteurization (heating milk at 62.5 ± 0.5°C for 30 min), high-temperature short-time pasteurization (HTST, in this instance performed at 62°C for 5 s) and/or freeze-thawing of BM was applied by 89 (58%) of participating units (Table 1). For the freeze-thawing method milk was frozen with a median freezing time of 1 day (range 0.5–14) at a median temperature of −20°C (range −80 to −8). Discontinuation of BM treatment for CMV inactivation or bacterial count reduction and the initiating of breastfeeding of CMV seropositive mothers were considered based upon the postmenstrual age and the actual body weight of the infant (Figure 1).

**Table 1:** Methods applied for CMV inactivation and bacterial count reduction in breast milk.

	**Total (*n* = 152) *n* (%)^#^**	**Germany (*n* = 126) *n* (%)^#^**	**Level III (*n* = 92) *n* (%)^#^**	**Level II (*n* = 27) *n* (%)^#^**	**Switzerland (*n* = 10) *n* (%)^#^**	**Level III (*n* = 6) *n* (%)^#^**	**Level II (*n* = 4) *n* (%)^#^**	**Austria (*n* = 16) *n* (%)^#^**	**Level III (*n* = 8) *n* (%)^#^**	**Level II (*n* = 7) *n* (%)^#^**
CMV inactivation	89 (58)	74 (58)	61 (66)	13 (48)	3 (30)	3 (50)	0 (0)	12 (75)	6 (75)	6 (86)
Holder-Pasteurization	53 (60)	44 (60)	39 (62)	6 (46)	2 (67)	2 (67)	n.a.	7 (58)	5 (83)	2 (23)
High-temperature short-time pasteurization	11 (12)	10 (14)	9 (15)	1 (8)	1 (23)	1 (13)	n.a.	0 (0)	0 (0)	0 (0)
Freeze-thawing method	25 (28)	20 (27)	13 (21)	6 (46)	0 (0)	0 (0)	n.a.	5 (42)	1 (17)	4 (67)
Bacterial count reduction^¶^	28 (17)	22 (17)	17 (18)^§^	5 (19)	2 (20)	2 (33)	0 (0)	4 (25)	3 (38)	1 (14)
Holder-Pasteurization	23 (82)	18 (86)	14 (82)	4 (80)	1 (50)	1 (50)	n.a.	4 (100)	3 (100)	1 (100)
High-temperature short-time pasteurization	3 (11)	1 (0.5)	1 (6)	1 (20)	1 (50)	1 (50)	n.a.	0 (0)	0 (0)	0 (0)

# *Denominator: Units participating in total, in each country and per level of neonatal care within each country*.

§ *Freeze-thawing method for bacterial count reduction: n = 1*.

¶ *Missing numbers = answers not given*.

*CMV = cytomegalovirus; Holder-Pasteurization = 62.5°C, 30 min; High-temperature short-time pasteurization = 62°C, 5 s*.

*n.a., not applicable*.

### Bacterial count screening and reduction

Sixty-five units (43%) routinely screened for bacterial BM colonization, either if BM was expressed at home (*n* = 7), expressed at the unit (*n* = 2) or both (*n* = 56). BM was pasteurized by 28 out of 65 units and/or discarded by 48 out of 65 units if bacterial counts exceeded pre-defined thresholds. In general, threshold levels varied considerably between units (Table 2). Bacterial count reduction was performed by Holder-pasteurization (*n* = 20) or HTST pasteurization (*n* = 3) (Table 1). Again, the duration of BM treatment for bacterial count reduction was depending on the postmenstrual age or the actual body weight of the infant (Figure 1).

**Table 2:** Bacterial count thresholds for pasteurization **(A)** and discarding **(B)** of breast milk intended for premature infants < 32 weeks postmenstrual age or body weight < 1,500 g.

**Evidence of**	**Replies *n* (%)**	**No pasteurization needed**	**Bacterial count limits (colony-forming units/mL)**				
			**>0**	**≥10^2^**	**≥103**	**≥10^4^**	**≥10^5^**
**(A) PASTEURIZATION OF BREAST MILK FOR BACTERIAL COUNT REDUCTION ACCORDING TO POSITIVE CULTURE RESULTS**							
Skin commensals	27 (100)	12 (44)	0 (0)	0 (0)	0 (0)	5 (19)	10 (37)
Staphylococcus aureus	27 (100)	5 (19)	7 (26)	1 (4)	7 (26)	4 (15)	3 (10)
Gram-negative bacteria	26 (100)	4 (15)	14 (54)	0 (0)	5 (19)	1 (4)	2 (8)
Bacillus cereus	24 (100)	8 (33)	8 (33)	0 (0)	4 (17)	2 (8)	2 (8)
**(B) DISCARDING OF BREAST MILK DUE TO BACTERIAL CONTENT ACCORDING TO POSITIVE CULTURE RESULTS**							
Skin commensals	48 (100)	30 (63)	0 (0)	3 (6)	4 (8)	2 (4)	9 (19)
Staphylococcus aureus	48 (100)	13 (27)	9 (19)	6 (13)	4 (8)	3 (6)	13 (27)
Gram-negative bacteria	48 (100)	12 (25)	12 (25)	5 (10)	5 (10)	6 (13)	8 (17)
Bacillus cereus	44 (100)	14 (32)	13 (30)	1 (2)	6 (14)	2 (4)	8 (18)

### Nutrient analysis and breast milk fortification

Only sixteen units (12%) were performing BM nutrient analysis using a bedside infrared analyser. Six of those regularly measured the BM nutrients content as part of a nutritional regime, five units occasionally and five units within clinical trials. Fortification in addition to standard multicomponent fortifier was performed by 75/135 units (58%). Additional protein was added to already fortified BM by 50%, lipids by 38% and carbohydrates by 15% of units. The decision on which component should be added was not revealed by our survey. In three units, fortification was adapted after nutrient analysis of mothers' own milk (targeted fortification) or according to the periodic determinations of the infant's blood urea nitrogen in four units (adjusted fortification).

### Organizational and departmental structures

Organizational details for the location of BM handling and storage as well as designated work force and responsibilities for BM handling are given in Table 3. BM was stored at a median temperature of −20°C (range −8 to −33) for a median of 6 months (range 0.07–8) before being discarded.

**Table 3:** Organizational details of breast milk handling.

Location of frozen BM storage	Replies *n* (%)	Neonatal ward	Milk kitchen (separate from neonatal ward)	Milk bank (also preparing donor milk)	Other location (e.g., hospital main kitchen, with parents)
	136 (100)	71 (54)	52 (38)	9 (7)	4 (3)
Location of BM preparation (thawing, pasteurization, portion)	Replies *n* (%)	Neonatal ward	Milk kitchen (separate from neonatal ward)	Milk bank (also preparing donor milk)	Other location (e.g., main hospital kitchen)
	139 (100)	70 (50)	60 (43)	9 (6)	4 (0)
BM is prepared by^*^	Replies *n* (%)	Nursing staff	Designated milk bank personnel	Main hospital kitchen personnel	Other provider (dietician, nutritionist)
	139 (100)	78 (56)	60 (43)	1 (0.7)	4 (3)
BM handling under the direction of	Replies *n* (%)	Nursing staff	Medical team	Other (dietician, nutritionist, IBCLC)	Not explicitly assigned
	138 (100)	92 (67)	21 (15)	2 (1)	23 (17)

* *Multiple replies possible*.

*BM, breast milk*.

### Breast milk administration errors

One hundred twenty-five units (82%) replied when queried about the incidence of BM administration errors per year with at least one incident of feeding BM to another than the intended infant in 91/125 units (73%). This relates to 66% of level III, 50% of level II, and 29% of level I units. There were either no cases of BM administration error (*n* = 34), 1–5 errors per year (*n* = 78), 6–10 per year (*n* = 9) or more than 10 per year (*n* = 4) reported.

## Discussion

Our survey reveals wide differences concerning many aspects of BM handling within participating units.

CMV inactivation of BM has been promoted to reduce the incidence of BM transmitted CMV infection (11). According to our survey, rates of maternal CMV screening and of CMV inactivation in mothers' own milk are comparable if not increased compared to corresponding data collected nearly a decade ago within the same countries (8) and appear to be more prevalent than in others (9, 10). CMV seropositive mothers' BM treatment for CMV inactivation was on average commenced on day 4 by the participants consistent with the occurrence of CMV in BM after the first week of lactation (12). Interestingly, there appears to be an agreement amongst participant concerning the postmenstrual age and body weight required to terminate BM treatment for CMV inactivation (and/or bacterial count reduction). However, CMV transmission rates, incidence of clinical signs of infection or sepsis and the impact of a postnatal CMV infection on neonatal short- and long-term outcomes remain controversial (13). While some data concerning neurocognitive development or hearing function point toward an unaffected outcome after BM transmitted CMV infection others suggest long-term neuropsychological sequelae (14–19). Therefore, the relevance of BM transmitted CMV infection and thus the role of CMV inactivation remains uncertain and official recommendations are not consistent. The Austrian Society of Pediatrics and Adolescent Medicine recommends freeze-thawing of colostrum and BM of CMV seropositive mothers for all infants < 32 weeks gestational age (20). The national German Breastfeeding Committee does not recommend pasteurization for CMV inactivation due to insufficient data (21) and official recommendations for Switzerland are not available.

A substantial number of neonatal units are performing routine BM cultures to assess an apparent need for bacterial count reduction or discarding of BM. Indeed, there are several reports of sepsis and/or death caused by BM transmitted bacteria published (22). However, there was no association between BM pathogens and the subsequent pathogen causing an infant's illness in a single center analysis of 813 BM cultures of 209 infants (3). To the best of our knowledge, no data from observational studies or randomized trials are available to support bacterial count reduction in mother's own BM to reduce neonatal morbidity. In fact, a trend toward an increased rate of necrotizing enterocolitis was observed in an Austrian neonatal unit after its unit policy was changed in favor of pasteurization of BM (23). Furthermore, in their randomized controlled trial Cossey et al. noted a trend toward an increased rate of late onset sepsis in infants fed pasteurized BM compared to those fed raw BM. However, results of this trial need to be interpreted with caution since BM containing any gram-negative organisms, *Staphylococcus aureus* or enterococci, was withheld and replaced by formula (24). The loss of humoral and cellular mediated immunological, antibacterial and enzymatic BM properties due to pasteurization may have an impact on BM mediated neonatal immunocompetence and on above mentioned observations (25). HTST pasteurization may increase protein retention rates compared to Holder-pasteurization but data concerning antibacterial efficacy of HTST pasteurization are controversial (25, 26). Because there is no robust evidence to guide the assessment of a safe bacterial load of BM when feeding premature infants, any distinction between BM colonization and BM contamination remains arbitrary (27). Therefore, interpretation of bacterial BM counts as well as bacterial spectrum differed widely, 30 different cut off values for bacterial content indicating BM treatment or discarding were reported in our survey. A survey of nine neonatal units from Belgium and Luxembourg showed similar inconsistent results (10). The German Breastfeeding Committee does not recommend pasteurization for bacterial count reduction (28). No recommendations for Switzerland and Austria are available. In conclusion, the role of routine BM cultures and bacterial count reduction remains uncertain.

Breast milk services were mostly headed by nursing staff members. In some units however, there was no explicit allocation of responsibility. This may prove unfavorable in terms of organizational management and liability. Only in the minority of units personnel was exclusively tasked with BM handling. In these cases, BM was mostly handled and stored not on the neonatal unit but in separate facilities. However, in most units regular nursing staff was tasked with BM handling next to their obligations as primary caregivers on the neonatal ward. Our survey revealed a high rate of BM administration errors throughout most units. Computerized provider order entry systems and adequate resource allocation may reduce BM administration errors (8).

Bedside BM nutrient analysis is performed in some units. Clinically relevant variations in results obtained from near-infrared compared to wet bench nutrient analysis were demonstrated and despite calibration adjustments concise near-infrared measurement of BM macronutrient content remains challenging (29, 30). Therefore, the Committee on Nutrition of the German Society for Pediatrics issued a statement against the indiscriminate use of human milk analyzers (31).

Standard fortification represents the predominant form of BM fortification. Fortification targeted according to BM nutrients content or adjusted to the infant's metabolic response (i.e., blood urea nitrogen levels) is rarely applied. But most units are adding additional proteins, lipids or carbohydrates to BM that has already been fortified with standard multicomponent fortifier, albeit on what basis remains unclear. Effects of increased osmolality need to be taken into account (32).

There are limitations to our survey. We did not inquire about the preferred feeding regimens if BM of CMV seropositive mothers was not pasteurized. The response rate to our survey was limited and varied between regions and countries, which may have influenced our results. However, comparable studies focused on specific BM handling aspects or included a limited number of units. The strength of our survey lies in the number of participating units within three different countries, providing insight into many different aspects of, to some extent, very diverse BM handling routines.

## Conclusions

There is a wide variability in most aspects of BM handling in the participating units. Despite limited evidence of clinical relevance, labor and cost intensive procedures are applied which may have an impact on BM quality. Evidence based data are needed to formulate reliable guidelines and strong recommendations for handling of human milk for premature infants.

## Ethics statement

This study was approved by the ethics committee of the Albert-Ludwigs-University of Freiburg, Germany (No. 484/16).

## Author contributions

DK conceived and designed the survey, contributed to data collection, analyzed data and wrote the first draft of the manuscript. SJ designed the survey, collected data, contributed to data analysis and reviewed the manuscript. CG designed the survey, contributed to data collection and reviewed the manuscript. HF contributed to data collection and reviewed the manuscript.

### Conflict of interest statement

The authors declare that the research was conducted in the absence of any commercial or financial relationships that could be construed as a potential conflict of interest.

## Abbreviations

BM: breast milk
CMV: Cytomegalovirus
HTST: High-temperature short-time.
