# Supplementary material for: Handling of Breast Milk by Neonatal Units: Large Differences in Current Practices and Beliefs
Source: Front Pediatr. 2018 Sep 4;6:235. doi: 10.3389/fped.2018.00235 (PMC6131667; doi:10.3389/fped.2018.00235)
Supplement: Supplementary file 1 [file Data_Sheet_1.PDF]

Handling of breast milk by neonatal units: Large differences in current practices and beliefs

Translated and adapted version of the stratified questionnaire. Conditional branching is not depicted.

Abbreviations: BM, breastmilk; CMV, cytomegalovirus.

Q1: What level of neonatal care are you providing?

a) Level III (highest level of neonatal care)
b) Level II
c) Level I
d) Other

Q2: How many inborn infants below 1500 g birth weight were treated in your unit in 2015?

Q3: Does your perinatal center perform antenatal maternal CMV screening until a certain postmenstrual age?

Q4: Do you perform CMV inactivation in BM under certain circumstances?

Q5: Until which postnatal body weight (not birth weight) of the infants do you perform CMV inactivation?

a) Not depending on body weight
b) < 1000 g
c) < 1250 g
d) < 1500 g
e) < 2000 g

Q6: Until which postmenstrual age of the infant do you perform CMV inactivation?

a) Not depending on postmenstrual age
b) 26 weeks
c) 28 weeks
d) 30 weeks
e) 32 weeks

Q7: Do you feed raw colostrum of CMV seropositive mothers?

a) No, colostrum will be subjected to CMV inactivation
b) Yes, until day 2 of life
c) Yes, until day 4 of life
d) Yes, until day 6 of life
e) Other

Q8: What methods are used for CMV inactivation in your unit?

a) Freeze/thawing
b) Holder-Pasteurisation (62.5 [+/- 0.5] °C /30 min)
c) Short-time pasteurisation (< 30 min)
d) None
e) Other

Q9: Which plateau time and -temperature are you using for short-time pasteurisation?

- Time (s):
- Temperature (°C):
- Others/comments:

Q10: For how long do you freeze BM for CMV inactivation at least? Please provide number of days, weeks or months.

- Days:
- Weeks:
- Months:
- Another timeframe:

Q11: Which of the following conditions is required for premature infants to be breastfed by CMV seropositive mothers in your unit?

- Postmenstrual age (weeks):
- Body weight (g):
- Others/comments:

Q12: Do you perform a bacterial screening of BM in your unit under certain circumstances?

a) Yes
b) No

Q13: Which of the following milk samples will undergo bacterial screening?

a) Expressed in the hospital
b) Expressed at home
c) Both

Q14: Do you perform bacterial count reduction in BM in your unit?

a) Yes
b) No, contaminated milk will be discarded if a certain bacterial BM threshold level is exceeded

Q15: What is the bacterial cut off level for the indication of breast milk treatment according to the respective bacteria?

Evidence of	Not tested for	No pasteurisation needed	Bacterial count limits (colony-forming units/mL)				
			> 0	≥ 10^2	≥ 10^3	≥ 10^4	≥ 10^5
Skin commensals	○	○	○	○	○	○	○
Staphylococcus aureus	○	○	○	○	○	○	○
Gram-negative bacteria	○	○	○	○	○	○	○
Bacillus cereus	○	○	○	○	○	○	○

Q 16: Until which postnatal body weight of the infant (not birth weigth) do you perform a bacterial count reduction?

a) Not depending on body weight
b) < 1000 g
c) < 1250 g
d) < 1500 g
e) < 2000 g

Q 17: Until which postmenstrual age do you perform a bacterial count reduction?

a) Not depending on postmenstrual age
b) 26 weeks
c) 28 weeks
d) 30 weeks
e) 32 weeks

Q18: What methods are used for bacterial count reduction in your unit?

a) Holder-Pasteurisation (62.5 [+/- 0.5] °C /30 min)
b) Short-time pasteurisation (< 30 min)
c) Freeze/thawing

Q19: What is the bacterial cut off level for the indication of discarding of breast milk according to the respective bacteria?

Evidence of	Not tested for	No pasteurisation needed	Bacterial count limits (colony-forming units/mL)				
			> 0	$\geq 10^2$	$\geq 10^3$	$\geq 10^4$	$\geq 10^5$
Skin commensals	○	○	○	○	○	○	○
Staphylococcus aureus	○	○	○	○	○	○	○
Gram-negative bacteria	○	○	○	○	○	○	○
Bacillus cereus	○	○	○	○	○	○	○

Q20: Where is frozen BM stored within your unit?

a) Neonatal ward/NICU
b) Separate “milk kitchen”
c) Breast milk bank (also preparing donor milk)
d) (Main) hospital kitchen
e) Others

Q21: Please detail the temperature and duration of storage for BM.

- Temperature (°C):
- Duration (months):
- Others/comments:

Q22: At what location are the actual breastmilk handling procedures (defreeze, portion…) performed?

a) Neonatal ward/NICU
b) Separate “milk kitchen”
c) Breast milk bank (also preparing donor milk)
d) (Main) hospital kitchen
e) Others

Q23: Who is performing the actual breastmilk handling procedures (defreeze, portion…)?

a) Nursing staff (also taking care of patients)
b) Designated personnel for “milk kitchen” or milk bank
c) Main hospital kitchen personnel
d) Dietician/nutritionist
e) Lactation consultant
f) Others

Q24: Who is in charge of the “milk kitchen” or the breast milk bank or milk bank services for the NICU?

a) Nursing staff (explicitly, but not necessarily exclusively designated)
b) Medical staff (explicitly, but not necessarily exclusively designated)
c) No one is explicitly in charge
d) Others

Q25: Do you perform BM nutrients analysis using bedside near infrared analyzers?

a) No
b) Regurlaly, as part of a nutritional regimen
c) In individual cases
d) In clinical trials
e) Others

Q26: Do you add nutrients to BM in addition to standard fortification using multicomponent fortifier (e.g. FM85)?

a) No
b) Protein
c) Lipids
d) Carbohydrates
e) Others

Q27: How do you proceed in this regard?

a) Individual decision
b) Target fortification (after BM nutrient analysis)
c) Adjusted fortification (after BM nutrient analysis and infant’s metabolic response)
d) Others

Q28: What is the incidence of breast milk administration errors in your unit per year?

a) None recorded
b) > 10 per year
c) 6-10 per year
d) 1-5 per year
e) Others
